# Generating *Shigella* that internalize into glioblastoma cells

**DOI:** 10.3389/fonc.2023.1229747

**Published:** 2023-11-23

**Authors:** Austin Shipley, Gabriel Frampton, Bryan W. Davies, Benjamin J. Umlauf

**Affiliations:** ^1^ Department of Neurosurgery, Dell Medical School, The University of Texas at Austin, Austin, TX, United States; ^2^ Department of Molecular Biosciences, The University of Texas at Austin, Austin, TX, United States; ^3^ John Ring LaMontagne Center for Infectious Diseases, The University of Texas at Austin, Austin, TX, United States; ^4^ Mulva Clinic for the Neurosciences, The University of Texas at Austin, Austin, TX, United States

**Keywords:** *Shigella*, glioblastoma, drug delivery, microbial factories, myristoylation

## Abstract

**Introduction:**

The use of microorganisms as drug delivery systems to treat cancer has expanded recently, including FDA approval of certain viruses as oncolytics. Microorganisms have several unique benefits compared to traditional pharmacologic agents including dose independence, the ability to produce therapeutic proteins locally within the tumor, and simplicity of administration. However, current microbial delivery systems such as AAV9 and herpes virus have limited cassette sizes, minimal cancer cell selectivity, and low innate cytotoxicity. To address these issues, we sought to generate a strain of *Shigella flexneri* to selectively internalize into glioblastoma (GBM) brain tumor cells as an initial step to generating a bacterial-based drug delivery system.

**Methods:**

We generated *S. flexneri* that selectively internalize into GBM cells using iterative co-cultured assays.

**Results:**

After 50 rounds of co-culture, the new strain infected 95 percent of GBM cells in 2 hours. GBM-infecting Shigella demonstrate a 124-fold preference for internalizing in nine different GBM cell lines compared to Normal Astrocytes (NA) controls. Additionally, we developed an in-cell western to identify GBM-infecting Shigella clones that preferentially internalize in patient samples without iterative co-culture. Finally, we demonstrate internalization into GBM cells is mediated via a factor modified by myristoylation.

**Discussion:**

In conclusion, here we present a novel bacterial platform that preferentially internalizes in brain tumor cells. This system provides numerous potential benefits over current interventions and other microbial strategies for treating brain tumors.

## Introduction

Microorganism-based drug delivery systems are emerging as a promising approach to treating solid tumors (1–4). Most strategies focus on using a virus (for example, herpes simplex virus) to directly lyse malignant cells and/or edit the cancer genome through gene therapy (4–11). While current microbial delivery methods have the potential to radically improve outcomes compared to traditional chemotherapy, inherent features of these viruses such as small cassette size, low cancer cell selectivity, and poor innate cytotoxicity have limited the use of this class of therapeutics (11). To address these issues, we generated an intracellular bacterium to serve as the basis of a drug delivery system to use as a novel therapeutic platform to treat brain tumors in the future (12–14).

Glioblastoma (GBM) is the most common malignant, primary brain tumor observed in adults, and patients diagnosed with GBM demonstrate a median survival of only 15 months (15, 16). The standard of care for GBM is gross total surgical resection, if possible, followed by radiation and chemotherapy. Even with complete surgical resection of the tumor and adjuvant chemotherapy, these tumors reoccur, ultimately resulting in mortality (15–19).

The basis for our system is *Shigella flexneri*, a gram-negative intracellular bacterium similar to the viruses discussed above. Importantly, *Shigella* has a type 3 secretion system capable of administering toxic proteins into the host cell cytosol (12, 20, 21),, and multiple tools exist to improve the safety of the bacterium. Further, *Shigella* has an unlimited cassette space to encode therapeutic proteins (22–24). Thus, this system has multiple potential benefits compared to current oncolytic virus platforms. Additionally, a recent report demonstrated that co-administration of attenuated *Salmonella* and neutrophil-derived doxorubicin nanoparticles significantly reduced the tumor burden in an orthotopic, murine model of glioblastoma (25). Here, we examined the safety profile of the parental and GBM-infecting *S. flexneri* strain, generated *Shigella* that selectively internalizes into GBM cells via iterative co-culture, and identified that a myristoylation enzyme is essential for internalization into GBM cells. In summary, this manuscript describes the generation and initial testing of the GBM-infecting *Shigella* platform that can be used in the future as a drug delivery system to treat brain tumors post-surgical resection.

## Materials and methods

### Origin and culture of *S. flexneri* strain 2475 serotype 2a

*S. flexneri* strain 2475 serotype 2a was grown from frozen Davies lab stocks that were originally a gift from Shelley M. Payne. Unique *Shigella* culture conditions were used for each assay described below. Generally, *Shigella* was streaked out from frozen stocks onto Tryptic Soy Broth (TSB) (30 g/L) + agar (15 g/L), and Congo Red (100 mg/L) sterile plates and incubated overnight at 30°C or 37°C. Individual colonies were expanded in sterile Tryptic Soy Broth (30 g/L) using a shaking incubator (250 rpm) at 30°C.

### Shiga toxin protein detection

At 24 hours prior to testing, *Shigella* was streaked out from the frozen stock onto TSB (30 g/L) + Congo Red (0.01% w/v) plates and incubated overnight at 37°C. Colonies were picked and grown for approximately 2 hours (OD_650 = _0.6) (13). Samples were tested according to the manufacturer’s protocol for ImmunoCard STAT!^®^ EHEC (Cat#: 751630). Band intensity was measured using ImageJ.

### Detecting shiga toxin DNA

A single *Shigella* colony was picked using a pipet tip and resuspended in 20 μL nuclease-free water. A colony was incubated for 10 min at 95°C and then centrifuged at 1,000 rcf for 1 min to remove membrane components.

Supernatant at a volume of 5 μL was used as the template for a PCR assay to quantify the presence of DNA encoding for Shiga toxin(s). Potential DNA regions were amplified with the Phusion taq polymerase kit (HF buffer, 20 cycles) using previously described primers that are specific for Shiga toxin 1 (Stx1 – primer pair: ATGTCAGAGGGATAGATCCA and TATAGCTACTGTCACCAGACAAT), Shiga toxin 2 (Stx2 – primer pair: AGTTCTGCGTTTTGTCACTGTC and CGGAAGCACATTGCTGATT), or positive control virulence factor F (virF – primer pair: AGCTCAGGCAATGAAACTTTGAC and TGGGCTTGATATTCCGATAAGTC) (26). Bands were identified by staining gel with SYBR Green dye (1:10,000 dilution) and imaging using a blue light transilluminator. Band intensity was quantified using the publicly available ImageJ software analysis suite.

### MsbB gene deletions

Genes were deleted as previously described using standard Lambda red recombineering methods with minor modifications (22, 27). *S. flexneri* was streaked onto TSB agar plates with 0.01% Congo Red and grown at 37°C overnight. The next day, 96 red colonies of *Shigella* were picked using a filtered p200 pipet tip. Each colony was added into 400 μL of TSB in a deep-well block plate and incubated at 37°C, 250 rpm, until an OD_650_ of 0.6 was reached (approximately 2.5 to 3 hours). *Shigella* suspensions were combined and pelleted into a 50-mL conical tube. After washing three times with 25 mL of 10% glycerol, *Shigella* was resuspended in 5 mL of 10% glycerol. *Shigella* was electroporated with 200 ng of pRedTKI plasmid DNA (Addgene Plasmid #51628) and recovered in TSB for 1 hour at 30°C, followed by plating on TSB + 50 μg/mL Kan agar plates with 0.01% Congo Red overnight. The pRedTKI plasmid introduces the Lambda red genes necessary for homologous recombination along with conferring Kan resistance.

*Shigella* + pRedTKI colonies were picked and incubated at 30°C, 250 rpm, until an OD_650_ of 0.6 was reached (approximately 3 hours); during the last 30 min of incubation, l-arabinose was added at 10 mmol/L to induce the Lambda red recombination genes along with 50 μg/mL kanamycin to maintain transmission of the pRedTKI plasmid. Bacteria were pelleted in a 50-mL conical tube by centrifugation at 4,000 × *g*, transferred to a 1.5-mL Eppendorf tube, washed three times with 500 μL of 10% glycerol, and resuspended in 50 μL of 10% glycerol. Using an electroporation cuvette with a 2 mm gap, *Shigella* were transfected via electroporation with 200 ng of I-SceI-flanking resistance cassette with 70-bp homology adjacent to the gene to be deleted. The cassette for MsbB1 was as follows: TGGTGCGGGGCAAGTTGTGCCGCTACACTATCACCAGATTGATTTTTGCCTTATCCGAAACTGGAAAAGCTAGGGATAACAGGGTAATATGGAGAAAAAAATCACTGGATATACCACCGTTGATATATCCCAATGGCATCGTAAAGAACATTTTGAGGCATTTCAGTCAGTTGCTCAATGTACCTATAACCAGACCGTTCAGCTGGATATTACGGCCTTTTTAAAGACCGTAAAGAAAAATAAGCACAAGTTTTATCCGGCCTTTATTCACATTCTTGCCCGCCTGATGAATGCTCATCCGGAATTACGTATGGCAATGAAAGACGGTGAGCTGGTGATATGGGATAGTGTTCACCCTTGTTACACCGTTTTCCATGAGCAAACTGAAACGTTTTCATCGCTCTGGAGTGAATACCACGACGATTTCCGGCAGTTTCTACACATATATTCGCAAGATGTGGCGTGTTACGGTGAAAACCTGGCCTATTTCCCTAAAGGGTTTATTGAGAATATGTTTTTCGTCTCAGCCAATCCCTGGGTGAGTTTCACCAGTTTTGATTTAAACGTGGCCAATATGGACAACTTCTTCGCCCCCGTTTTCACCATGGGCAAATATTATACGCAAGGCGACAAGGTGCTGATGCCGCTGGCGATTCAGGTTCATCATGCCGTTTGTGATGGCTTCCATGTCGGCAGAATGCTTAATGAATTACAACAGTACTGCGATGAGTGGCAGGGCGGGGCGTAATAGGGATAACAGGGTATAAAAGCCTCTCGCGAGGAGAGGCCTTCGCCTGATGATAAGTTCAAGTTTGCTTCAGAATATTCGAAATCT.

The cassette for MsbB2 was as follows: AATTAAGGTTAGATGTATTCTCTGAATAAAATATTAATGATGATTATGGTAGGGGCATTCGCACTAAATATAGGGATAACAGGGTAATATGGAGAAAAAAATCACTGGATATACCACCGTTGATATATCCCAATGGCATCGTAAAGAACATTTTGAGGCATTTCAGTCAGTTGCTCAATGTACCTATAACCAGACCGTTCAGCTGGATATTACGGCCTTTTTAAAGACCGTAAAGAAAAATAAGCACAAGTTTTATCCGGCCTTTATTCACATTCTTGCCCGCCTGATGAATGCTCATCCGGAATTACGTATGGCAATGAAAGACGGTGAGCTGGTGATATGGGATAGTGTTCACCCTTGTTACACCGTTTTCCATGAGCAAACTGAAACGTTTTCATCGCTCTGGAGTGAATACCACGACGATTTCCGGCAGTTTCTACACATATATTCGCAAGATGTGGCGTGTTACGGTGAAAACCTGGCCTATTTCCCTAAAGGGTTTATTGAGAATATGTTTTTCGTCTCAGCCAATCCCTGGGTGAGTTTCACCAGTTTTGATTTAAACGTGGCCAATATGGACAACTTCTTCGCCCCCGTTTTCACCATGGGCAAATATTATACGCAAGGCGACAAGGTGCTGATGCCGCTGGCGATTCAGGTTCATCATGCCGTTTGTGATGGCTTCCATGTCGGCAGAATGCTTAATGAATTACAACAGTACTGCGATGAGTGGCAGGGCGGGGCGTAATAGGGATAACAGGGTAATAATTATAAAGTACAGGTATTTCCACTAGTTGTTTCTTACAGGTTACCAATCGAAACACATCCCCCTTCCG.

The primer pair for identifying MsbB1 was TTGAACTTATCATCAGGCGAAGGCCTC and CGGCTTTTTTTATTTGGTGCGGGG, and that for MsbB2 was CTGCTATCCGCTCTTTGGATGCA and CTACACAGTCCTCCGTGCCAA. Electroporated *Shigella* was allowed to recover in Super Optimal broth with Catabolite repression (SOC) for 3 hours at 30°C and plated on TSB + 50 μg/mL Kan + 12.5 μg/mL chloramphenicol (Cam) agar plates with 0.01% Congo Red overnight.

To complete the gene deletion protocol, a single colony of *Shigella* + pRedTKI + Cam resistance cassette was incubated at 30°C, 250 rpm, until an OD_650_ of 0.6 (approximately 3 hours) in 3 mL TSB + 50 μg/mL Kan + 20 mmol/L isopropyl β-d-1-thiogalactopyranoside (IPTG) to induce I-SceI expression to excise the Cam resistance gene. A sample of liquid culture was seeded onto TSB + 50 μg/mL Kan + agar plates with 0.01% Congo Red and incubated overnight. Modifications were verified by PCR.

### Internalization assay

#### Tissue culture

GBM cell lines were obtained from Dr. John Kuo (GSC 112, 15, 109, 114, 124, 113, 115, and 99) or ATCC (U-251 MG, Cat#: 09063001, Lot#: 17K073). Normal astrocytes were purchased from Lonza (Cat#: CC-2565) and grown according to the manufacturer’s protocol without antibiotics. Mammalian cells were grown in tissue culture-treated T25 or T75 flasks in low-glucose Dulbecco’s modified Eagle’s medium (DMEM) with 10% fetal bovine serum (FBS), GlutaMAX, and 1% sodium pyruvate (without pen/strep) (28–30). Cells were split at 1:3 using Accutase (Innovative Cell Technologies, San Diego, CA, USA; Cat#: AT104) to remove cells from tissue culture plastic when >80% confluent. Cells were washed two times with complete media before replating. Neurons were harvested from rats using a typical protocol and maintained using a previously described culture protocol (31). Briefly, the hippocampus from mice was isolated via dissection, placed in a digestion solution consisting of l-cysteine, NaOH, CaCl_2_, DNAse I, EDTA, and papain for 35 min, and then inactivated in serum media. Cells then underwent trituration, were strained through a 100-μm cell strainer, and were centrifuged for 5 min at 200 × *g*, 4°C. The supernatant was aspirated, and cells were counted and replated at 750,000 cells/mL. Neurobasal media at a volume of 2 mL with B-27 supplement, GlutaMAX, and 1.25% FBS was added to a culture plate. Thirty percent of the media was replaced twice per week. After 3 days of culture, 5-fluoro-2′-deoxyuridine (FUDR) was added to remove dividing cells. All mammalian cells were cultured at 37°C, 5% CO_2_.

#### Co-culture assay

Mammalian cells were maintained in culture as described above. At 24 hours prior to co-culture assays, the media was changed without removing cells from the tissue culture flask. Cells grown to 80% confluency in a T25 flask were used for each round of co-culture.

*S. flexneri* was streaked on TSB agar plates with 0.01% Congo Red and grown at 37°C overnight. The next day, 96 red colonies of *Shigella* were picked using a filtered p200 pipet tip and added to individual wells of a deep-well block plate that contained 400 μL of TSB. The deep-well block plate was incubated at 37°C, 250 rpm, until an OD_650_ of 0.6 was reached (approximately 2.5 to 3 hours). A control well was used to estimate the *Shigella* growth rate. After reaching the mid-log phase (0.6 OD_650_), individual wells were pooled, and the *Shigella* was concentrated to 2 × 10^8^ cfu/mL (OD_650_ of 1.0 = 8.0 × 10^8^). Next, 250 μL of *Shigella* concentrate was added to ~5 mL of GBM, normal astrocyte (NA), or rNeuron media. *Shigella*-containing media at a volume of 5 mL was added to the T25 cell culture flask containing mammalian cells. The T25 culture flask was centrifuged at 200 × *g* for 10 min and then incubated at 37°C, 5% CO_2_, for 30 min. After 30 min, culture media was removed by aspiration. The T25 flask was washed four times using 5 mL of phosphate-buffered saline (PBS) for 1 min with gentle agitation. Next, 5 mL of mammalian culture media containing 20 μg/mL of gentamicin was added to the T25 flask. The T25 flask was incubated at 37°C, 5% CO_2_, for an additional 90 min. After 90 min, the media was aspirated, and cells were washed four times with PBS, 1 min per wash, with gentle agitation.

After the final wash, 1 mL of Accutase (Innovative Cell Technologies; Cat#: AT104) was added to the T25 flask. The T25 flask was incubated at 37°C, 5% CO_2_, until GBM, NA, or rNeuron cells detached from the plate. Mammalian cell membranes were mechanically disrupted by pulling the cell suspension through a 27Ga needle 10 times. The mammalian cell lysate was centrifuged at 1,000 × *g* for 2 min to pellet any membrane-associated (but not internalized) *Shigella*. Mammalian cell lysate at a volume of 20 μL was plated onto a TSB + 0.01% Congo Red agar plate and grown at 37°C overnight. Individual colonies were expanded and frozen in 25% glycerol or used directly for the next round of co-culture assays.

### Confocal fluorescence microscopy

#### Tissue culture

At 24 hours prior to internalization, GBM cells were grown to ~80% confluence using the protocol described above in the mammalian cell culture section on Poly-d-Lysine, No. 1.5 glass-bottom dishes (MatTek, Ashland, MA, USA; Cat#: P35GC-1.5-14-C).

An individual *Shigella* colony was picked, grown to the mid-log phase, and co-cultured with mammalian cells in the microscopy dish using the protocol described above in the co-culture assay section. After the final wash, cells were fixed with 4% paraformaldehyde (PFA) (Electron Microscopy Sciences, Hatfield, PA, USA; Cat#: 50-980-487) for 10 min and then washed three times with PBS.

#### For membrane + DNA imaging

PBS staining solution at a volume of 1 mL containing wheat germ agglutinin-fluorescein (WGA; 1:2,000, Vector Laboratories, Burlingame, CA, USA; Cat#: FL-1021) and Hoechst 33342 (1:800, Invitrogen, Carlsbad, CA, USA; Cat#: H3570) was added to the fixed cells and incubated for 15 min. Cells were washed three times with PBS. After the final wash, 1 mL of PBS was added to the dish so cells did not dry out during imaging. Cells were imaged using a 60× objective on a Nikon Eclipse Ti2 confocal microscope. Images were processed and analyzed using the publicly available ImageJ software package.

#### For *Shigella* antibody staining

Cells were permeabilized by adding 1 mL of 0.1% Triton X-100 in PBS for 5 min. Blocking buffer at a volume of 1 mL (PBS + 30 mg/mL bovine serum albumin (BSA) + 5% donkey serum + 0.1% Triton X-100) was added to each dish, and the cells were incubated at 4°C overnight. The next day, cells were washed three times with PBS. Next, cells were incubated with an anti-*Shigella* antibody (1:200, Abcam, Cambridge, UK; Cat#: ab65282) in 1 mL PBS + 30 mg/mL BSA + 0.1% Triton X-100 for 90 min at room temperature. After incubation, cells were washed three times with PBS. Next, cells were incubated with anti-rabbit Alexa Fluor 488 secondary (1:1,000, Invitrogen; Cat#: A21206) in PBS + 30 mg/mL BSA + 5% donkey serum + 0.1% Triton X-100 (1-mL total volume) for 60 min. Cells were washed three times with PBS. Next, cells were incubated with Phalloidin Dye 594 (1:1,000, Abnova, Taipei, Taiwan; Cat#: U0292) and Hoechst 33342 (1:800, Invitrogen; Cat#: H3570) in PBS (1-mL total volume) for 15 min. Cells were imaged using a 60× objective on a Nikon Eclipse Ti2 confocal microscope. Images were processed and analyzed using the publicly available ImageJ software package.

### In-cell western

#### Cell culture

At 24 hours prior to internalization, GBM or NA cells were grown to 80% confluence as described in the mammalian tissue culture section above on a tissue culture-treated, black well, clear bottom, 96-well tissue culture plate.

#### Co-culture

Individual *Shigella* colonies were picked and expanded as described above in a deep block 96-well plate. *Shigella* culture at a volume of 40 μL from a single well was added to 200 μL of GBM or NA media previously plated into each well of the clear-bottom, black, 96-well plate. The 96-well plate was centrifuged at 200 × *g* for 10 min and then incubated at 37°C, 5% CO_2_, for 30 min. Culture media was removed after 30 min by dumping the supernatant into a sterile glass dish containing bleach. The plate was washed four times with PBS using a similar method to remove the supernatant. Next, 200 μL of mammalian cell media containing 20 μg/mL gentamicin was added to each well. The plate was incubated at 37°C, 5% CO_2_, for 90 min. After incubation, the plate was washed four times with PBS. Cells were fixed by adding 200 μL of 4% PFA to each well and incubating for 10 min. The plate was washed three times with PBS. Cells were permeabilized by adding 200 μL of PBS containing 0.1% Triton X-100 to each well for 5 min. Blocking buffer containing 1% BSA and 1% donkey serum was added to each well, and the plate was incubated at 4°C overnight. The next day, the plate was washed three times with PBS. The plate was incubated with anti-*Shigella* antibody (1:200, Abcam; Cat#; ab65282) in PBS + 30 mg/mL BSA + 0.1% Triton X-100 (100 μL per well) for 90 min. The plate was washed with PBS three times. Finally, each well was incubated with IR_800_CW anti-rabbit secondary (1:10,000, LI-COR, Lincoln, NE, USA; Cat#: D20119-05) in PBS + 30 mg/mL BSA + 5% donkey serum + 0.1% Triton X-100 for 60 min. The plate was washed three times with PBS and imaged on a LI-COR Fc scanner using a 2-min medium resolution scan. A standard curve of *Shigella* was used on each plate to convert the IR_800_ signal to the number of bacteria per well. It was important to ensure that the moles of anti-*Shigella* antibody greatly exceeded the moles of *Shigella* to ensure the saturated binding assumption was valid in order to quantify the number of bacteria per well.

### Statistical methods

Data are generally presented as means with standard deviation. All validation experiments were conducted with a minimum of two independent replicates. For microscopy experiments, a minimum of three fields were quantified from at least two independent experiments. Significant differences between *Shigella* internalization in cell lines were determined using ANOVA with a 1% false discovery rate used as the threshold for significance. For microscopy assays, ANOVA was used to determine differences between groups with a 5% false discovery rate used as the threshold for significance.

## Results

### Overall scheme for generating *Shigella* that internalizes in brain tumor cells

**Figure 1:**
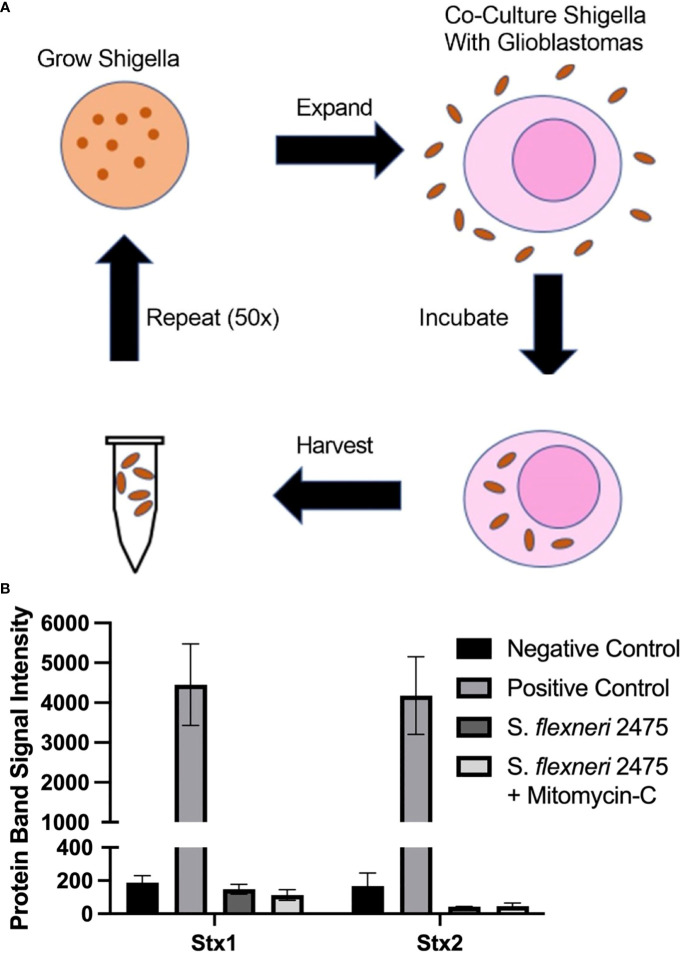
Scheme to generate GBM-infecting *Shigella* and verification of safety. **(A)** Scheme to select *Shigella flexneri* that preferentially internalize into glioblastomas. **(B)** The absence of Shiga toxin proteins in the parental strain of *Shigella* (*S. flexneri* 2475 serotype 2A) was measured using a Shiga toxin detection kit prior to co-culture. Lack of Shiga toxins was confirmed during normal growth conditions and with mitomycin C (Mito) induction. Band intensity corresponding to Stx1, Stx2, or controls is plotted for each group. GBM, glioblastoma.

As described in **Figure 1A**, we developed a scheme to identify *Shigella* clones that preferentially internalize into brain tumors using iterative co-culture assays. Briefly, *S. flexneri*, an intracellular bacterium, was streaked onto agar plates. Clones containing the virulence factor needed to survive inside mammalian cells were expanded and incubated with a brain tumor cell line, U-251 GBM. Bacteria that internalized into GBM cells were harvested and used for additional rounds of co-culture.

### Identification of an *S. flexneri* strain suitable for GBM internalization

In order to identify a *Shigella* strain that is safe to use as a therapeutic platform, we identified a strain that did not contain Shiga toxins. We probed *S. flexneri* strain 2475 serotype 2a for DNA regions encoding Shiga toxins using previously described primers. As expected, we did not observe a band at the expected size for the PCR product (**Supplementary Figure 1**) (26, 32, 33). Quantification of this region demonstrated 15- and 21-fold less intense signal compared to positive control PCR for virF. We also quantified levels of Shiga toxin 1 and 2 proteins in *S. flexneri* strain 2475 serotype 2a prior to co-culture using a rapid test commercially available detecting kit (**Figure 1B**). The parental *Shigella* strain was negative for both Stx1 and Stx2, relative to a positive control included in the kit, under normal conditions and when treated with mitomycin C to induce the lytic cycle of potential Stx-producing prophage. Finally, whole genome sequencing indicated there were no Shiga toxin encoding regions in the virulence plasmid or genome (**Supplementary Figure 4**).

### Generating *Shigella* that internalizes into a GBM cell line

**Figure 2:**
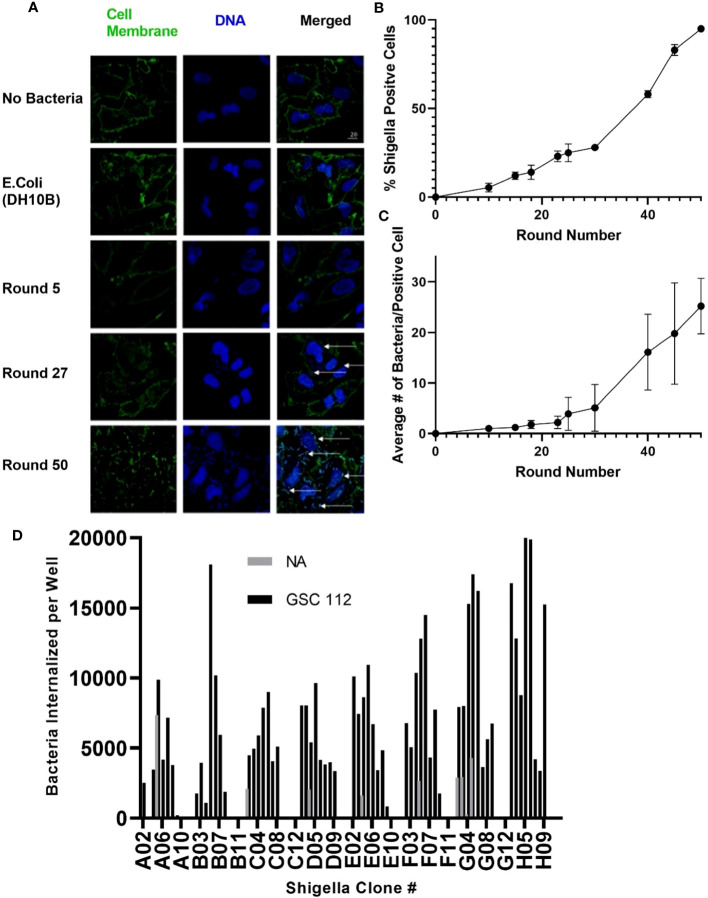
Generating *Shigella* that internalizes into a GBM cell line. *Shigella* was incubated in U-251 cells *in vitro*. Internalized *Shigella* was isolated and amplified. This process was repeated 50 times, as indicated by round number. **(A)** Confocal fluorescence microscopy of internalized *Shigella* in U-251 cells. *Shigella* was incubated with U-251 GBM cells, fixed, and stained with Hoechst 33342 for DNA (blue) and wheat germ agglutinin (WGA) for cell membranes (green). White arrows indicate individual *Shigella*. The scale bar indicates 20 μm. **(B)** The percentage of U-251 cells with internalized *Shigella* identified via microscopy is plotted over 50 rounds of co-culture with a minimum of three fields counted per round. **(C)** The mean number of *Shigella* rods per U-251 cell, with at least one *Shigella* rod inside the cell membrane, is plotted over 50 rounds of internalization. Three fields were counted for each round. **(D)** GBM-infecting *Shigella* were streaked, individual colonies expanded, and single colonies incubated with GSC 112 GBM cells. After incubation, the amount of internalized *Shigella* was quantified using an in-cell western. The number of *Shigella* internalized into GSC 112 and NA is plotted for 96 individual GBM-infecting *Shigella* clones. Clones exhibiting below detectable levels of the bacterial signal are represented as zero. GBM, glioblastoma; TSB, Tryptic Soy Broth; NA, normal astrocyte.

As described in **Figure 1**, *Shigella* clones were incubated with U-251 GBM cells. Internalized clones were selected and carried forward to the next round of co-culture. Thus, *Shigella* clones that internalized in GBM cells were enriched iteratively to generate a population of GBM internalizing *Shigella*. To monitor the degree of enrichment of *Shigella* clones that internalized in GBM cells during the selection process, we performed fluorescence microscopy and counted the number of *Shigella* internalized in GBM cells as the co-culture experiments progressed. As shown in **Figure 2A**, internalized *Shigella* appear as rod-like structures within the boundaries of the U251 GBM cell membrane (demarcated by wheat germ agglutinin) when stained with a DNA intercalating dye Hoechst 33342 (rendered blue). The number of GBM cells demonstrating an internalized bacterium increased after each round of *Shigella* co-culture, starting with 5% ± 2.5% in round 10 and enriching to 95% ± 1% by round 50 of co-culture. The percentage of GBM cells with an internalized bacterium was calculated by taking three randomly selected fields and dividing the total number of infected cells by the total number of U-251 cells present (**Figure 2B**). The number of individual *Shigella* bacteria present inside each infected cell also increased as the rounds of co-culture progressed (**Figure 2C**). In round 5, only one to two bacteria were present in an infected cell. By round 50, an average of 20 ± 5.5 bacteria were present within each infected GBM cell.

As demonstrated in **Figure 2D**, individual GBM-infecting *Shigella* clones were expanded, and then an internalization assay was performed using patient-derived GSC 112 GBM cells and NA cells. Cells were fixed, permeabilized, and subjected to a quantitative in-cell western using an anti-*Shigella* antibody and an IR_800_ signal as a readout. A *Shigella* standard curve was used to convert the fluorescent signal to the amount of *Shigella* in each well. Out of 96 total clones, 85 clones internalized below detectable levels in NA cells. All 96 clones demonstrated increased internalization in GSC 112 cells compared to NA cells. The average number of bacteria internalized into GSC 112 over the entire 96-well plate was 2.6 bacteria per GSC 112 cell. Values ranged from 10.367 to 0.06 bacteria per GSC 112 cell.

### GBM-infecting bacteria retain *Shigella* virulence plasmid

**Figure 3:**
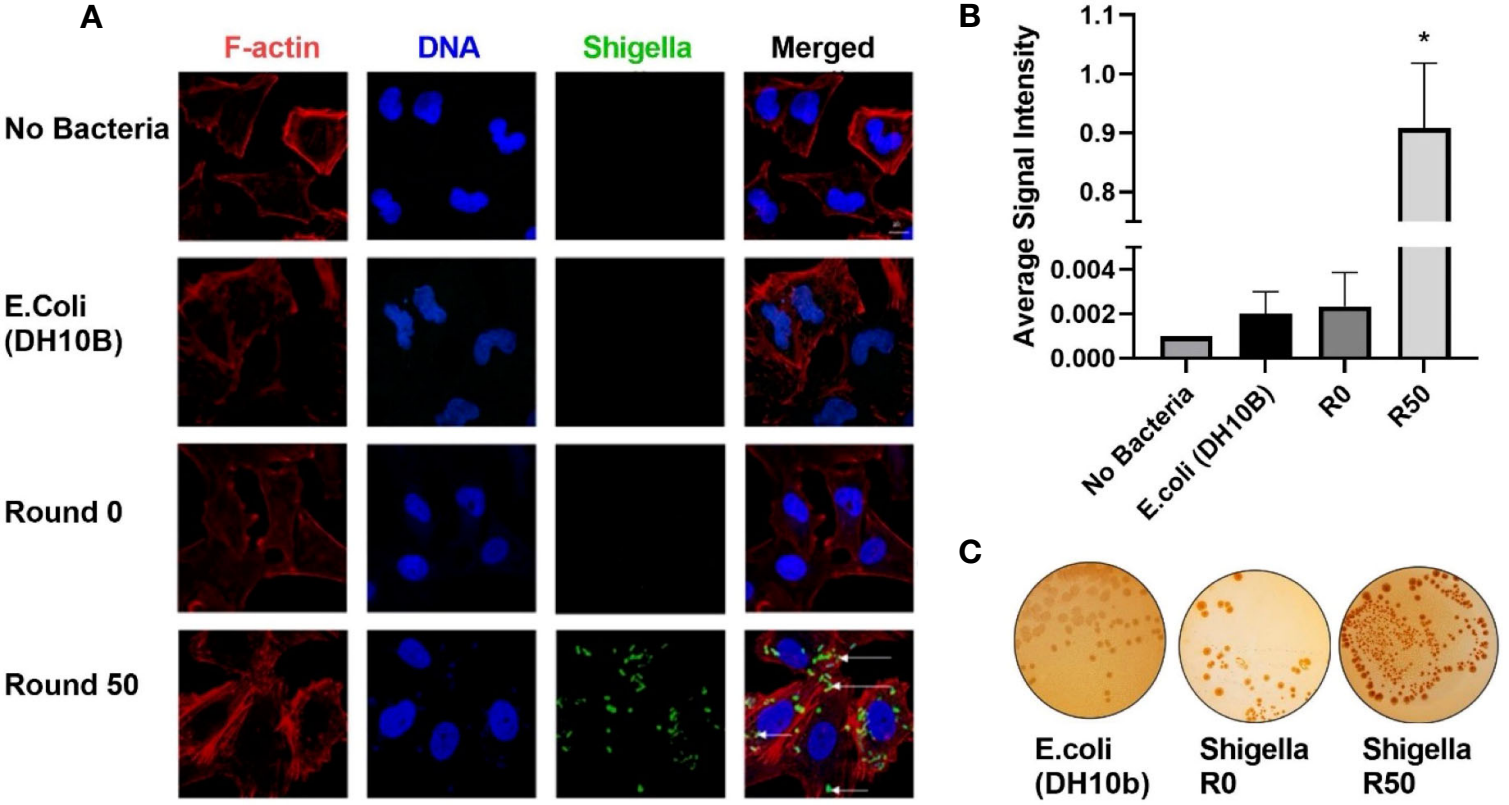
GBM-infecting *Shigella* retain *Shigella flexneri* phenotype. **(A)** GBM-infecting *Shigella* or control were incubated with U-251 GBM cells. Gentamycin and washing eliminated non-internalized bacteria. Cells were fixed, stained with Hoechst 33342 for DNA (blue), Phalloidin for F-Actin (red), anti-*Shigella* antibody (ab65282), and counterstained with anti-rabbit Alexa Fluor 488 (green) to visualize *Shigella* via fluorescence confocal microscopy. Scale bars indicate 20 μm, and white arrows highlight *Shigella*. **(B)** The average signal intensity of *Shigella* fluorescence is quantified and plotted (*p < 0.05 by ANOVA, n = 3 individual fields/group). **(C)** *Shigella* isolated from GBM cells was plated on TSB + Congo Red agar plates. Representative plates are shown. Red colonies demonstrate that GBM-infecting *Shigella* can still process Congo Red dye, indicating retention of virulence plasmid. GBM, glioblastoma.

U-251 cells were infected with round 50 GBM-infecting Shigella. Cells were fixed and then stained with an anti-Shigella antibody targeting the lipopolysaccharide (LPS), phalloidin-AF594, and Hoechst 33342 then imaged via confocal microscopy. The DNA rod-like structures observed inside GBM cells throughout this study co-localized with an anti-Shigella antibody (**Figure 3A**). Quantification of these images is presented in **Figure 3B**; a greater than 1,000-fold increase was observed in Shigella fluorescent signal in round 50 GBM-infecting Shigella (0.909 ± 0.110 rfu) compared to non-internalizing or other negative controls (non-internalizing (R0) = 0.002 ± 0.001 Escherichia coli 0.002 ± 0.001 no bacteria 0.001 ± 0.0). Additionally, GBM-infecting Shigella plated on TSB + 0.01% Congo Red agar plates uptake Congo Red (indicated by red colonies) similar to non-internalizing Shigella starting bacteria (positive control). A closely related E. coli (negative control) did not uptake the red dye as expected (**Figure 3C**). In addition, the Shiga toxin detection assay was repeated, and it is demonstrated in **Supplementary Figure 2** that GBM-infecting Shigella still did not express Shiga toxins under normal growth conditions or when induced with mitomycin C.

### GBM-infecting *Shigella* preferentially internalizes in GBM cells compared to normal astrocyte controls

**Figure 4:**
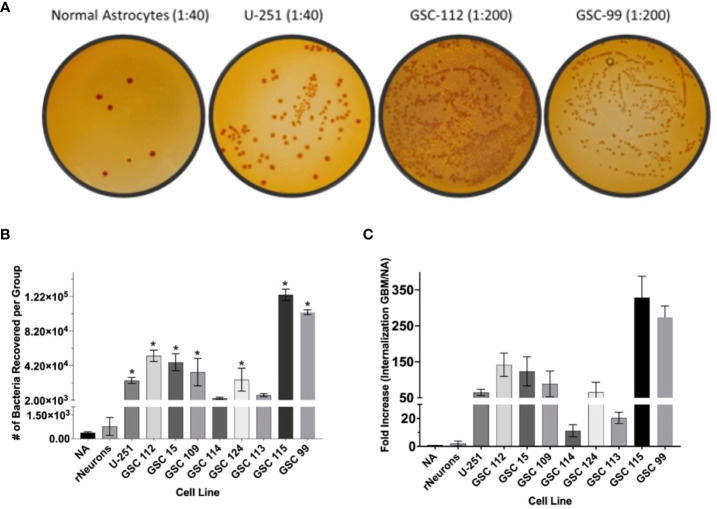
GBM-infecting *Shigella* preferential internalization into GBM cells compared to normal astrocyte controls. Internalized GBM-infecting *Shigella* was quantified in a panel of GBM cell lines, two unique normal astrocyte (NA) cell preparations, and rat neurons. **(A)** GBM-infecting Shigella was incubated with either GBM or normal astrocyte cells. Internalized Shigella was recovered and plated on TSB + Congo Red plates to quantify internalized bacteria. Representative images are demonstrated in panel **(A)** Dilutions depicted for NA and U-251 cells is 1/40. All GSC cell lines are diluted 1/200. **(B)** Shigella recovered from each cell line is plotted and compared to normal astrocytes (*p < 0.01 via multi-comparison corrected ANOVA). **(C)** The fold change of internalization for each GBM cell line compared to NA is plotted. The average fold change over all GBM cell lines was 123 ± 21.2 (standard error of the mean), and GSC 115 displayed the greatest fold change of 322 ± 34.3. GBM, glioblastoma; TSB, Tryptic Soy Broth.

Next, GBM-infecting *Shigella* was incubated with a panel of patient-derived GBM cell lines, as well as normal astrocytes and normal rat neurons, to quantify both the breadth and specificity of this platform to internalize in adult malignant brain tumors. Nine patient-derived glioblastoma stem cell (GSC) lines were incubated with GBM-infecting *Shigella*. Internalized *Shigella* was harvested and plated to count the number of internalized bacteria per group. GSC 115 showed the highest internalization at 123,800 bacteria/5 × 10^5^ GBM cells. To demonstrate the specificity of the platform, GBM-infecting *Shigella* were incubated with normal astrocytes and internalized bacteria counted. Approximately 383 bacteria internalized/~300 NA cells (two separate patient cell lines) were observed. **Figure 4A** shows the colony counts recovered after internalization for each GSC or NA cell line. A one-way ANOVA of the colony counts indicated that seven out of the nine GSC cell lines demonstrated significantly higher internalization (p < 0.01) compared to NA. **Figure 4C** depicts the fold change between each GSC cell line relative to NA. The average fold increase was 123 ± 110; GSC 115 demonstrated the highest internalization with a 373 ± 59.5-fold increase compared to NA.

### Whole genome characterization of GBM-infecting *Shigella*

We performed whole genome sequencing on GBM-infecting *Shigella* and compared the data to those of the parental strain. We identified 177 mutations in 46 genes (**Supplementary Figure 3** and **Supplementary Table 1**). Seventeen mutations were in genes related to metabolism/homeostasis, 13 genes were involved in DNA regulation, seven genes were involved in transcriptional regulation, six mutations were in genes associated with stress response, and three mutations occurred in genes with unknown function. Importantly, we identified no mutations that would affect the virulence or safety of the novel bacterium.

### Membrane anchored factor in novel *Shigella* strain mediates internalization in GBM cells

**Figure 5:**
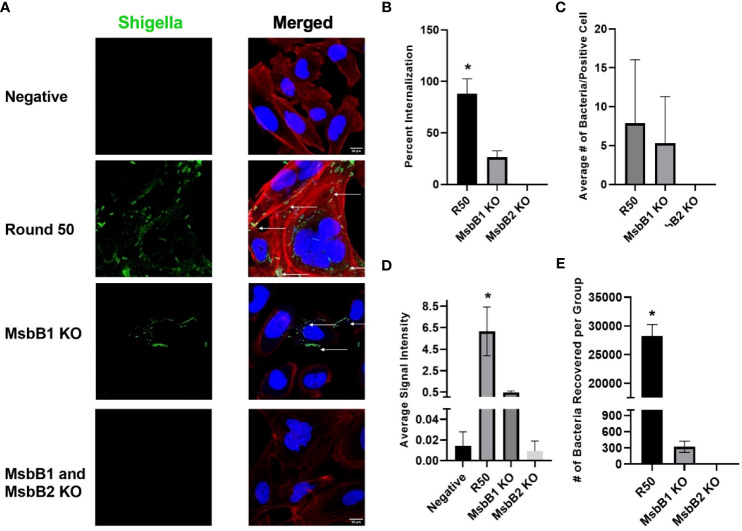
Characterization of GBM-infecting *Shigella*. **(A)** GBM-infecting *Shigella*, GBM-infecting *Shigella* MsbB1 and MsbB2 knock-out, and control *Shigella* were incubated with U-251 GBM cells. Gentamycin and washing eliminated non-internalized bacteria. Cells were fixed, stained with Hoechst 33342 for DNA (blue), Phalloidin for F-Actin (red), anti-*Shigella* antibody (ab65282), and counterstained with Alexa Fluor 488 (green) to visualize *Shigella* using confocal fluorescence microscopy. **(B)** The percentage of U-251 cells with internalized *Shigella* identified via microscopy is plotted. **(C)** The mean number of *Shigella* rods per U-251 cell, with at least one *Shigella* rod inside the cell membrane. **(D)** The average signal intensity of *Shigella* fluorescence is quantified and plotted (*p < 0.05 by ANOVA, n = 3 individual fields/group). **(E)** GBM-infecting *Shigella* or MsbB1 and MsbB2 knock-out *Shigella* are incubated with GBM cells. Internalized *Shigella* was recovered and plated on TSB + Congo Red plates to quantify internalized bacteria. GBM, glioblastoma; TSB, Tryptic Soy Broth.

We removed MsbB1 and MsbB2 myristoylation factors from the round 50 GBM-infecting *Shigella* using recombineering (**Supplementary Figure 4**). As expected from previous reports (34–37), removal of the myristoylation enzymes lowered the TLR4 detection relative to unmodified *Shigella* (p < 0.05 via ANOVA, **Supplementary Figure 5**). Interestingly, removing MsbB1 also decreased the recovery of *Shigella* that internalized into GBM cells from an average of 2.82 × 10^4^ to 320 bacteria per group (p < 0.05 via ANOVA). Removal of both MsbB1 and MsbB2 completely abrogated internalization to an average of <1 bacterium recovered. Confocal imaging of GBM cells treated with either R50, MsbB1 KO, or MsbB1 and MsbB2 KO demonstrate a significant decrease in cells internalizing *Shigella* (**Figure 5**). After MsbB1 was removed, the percentage of *Shigella*-positive cells dropped from 95% to 25% (p < 0.05 via ANOVA). Quantifying the fluorescent *Shigella* signal indicated that MsbB1 removal reduced the mean signal to 0.425 rfu compared to 6.16 rfu for R50 (~14.5-fold decrease in signal). Removal of both MsbB1 and MsbB2 resulted in 0% of cells with a *Shigella* internalized and a fluorescent signal of 0.009 rfu (~684-fold decrease). GBM cells that did demonstrate *Shigella* internalization after removal of myristoylation factor MsbB1 had similar numbers of internalized *Shigella* compared to R50. The growth curves of R50, MsbB1 KO, and MsbB1 and MsbB2 KO *Shigella* are presented in **Supplementary Figure 6**.

## Discussion

In this study, we demonstrated a method to generate *S. flexneri* clones that selectively internalize into GBM cell lines. We first selected a strain of *Shigella* that does not express Shiga toxins to improve the safety profile of the platform (12). After only 50 rounds of co-culture with GBM cells, *Shigella* clones demonstrated a high propensity for internalizing into GBM cells. We observed less than one cell per field with internalized *Shigella* after five rounds of co-culture compared to 95% of cells infected with multiple bacteria after 50 rounds of co-culture. GBM-infecting *Shigella* retained LPS surface markers and the virulence plasmid throughout the co-culture process. Finally, we developed an in-cell western screen to complement the co-culture assays to improve patient specificity of the platform without further co-culture assays. In-cell western screening provides the opportunity to personalize this platform quickly for each patient tumor or to identify an optimal GBM-infecting *Shigella* clone for GBM cell lines that cannot be reliably cultured for 50 rounds of co-culture assays.

Previous studies have used microorganisms as delivery vehicles for GBM and other brain tumors (1–8, 10, 11, 38). Most studies, including those advancing to clinical trials, have focused on using a virus to deliver a therapeutic to the tumor (4). While these studies have potential advantages over traditional chemotherapy, using a virus as a therapeutic factory presents challenges. Namely, most viruses have limited specificity for GBM cells, small cassette sizes, and safety concerns (39). GBM-infecting *Shigella* still replicates inside a mammalian cell, similar to the current virus strategies (14). However, the larger size and complexity of *Shigella* allow for rapid identification of clones that preferentially internalize into GBM (it typically takes hundreds of passages with a given cell type to change the tropism of a virus) (40, 41). A recent report described systemic administration of attenuated *Salmonella* to immunodeficient animals bearing orthotopic glioblastoma, resulting in *Salmonella* accumulation in hypoxic regions of the tumor. While this report relied on neutrophil-derived doxorubicin particles to mediate tumor regression, this study further demonstrates the propensity of bacteria to infect glioblastoma (25). Additionally, the large genome and DNA editing resources available for *Shigella* allow for any sized cassette to be permanently integrated into the genome (22, 27). Thus, almost any combination of therapeutic proteins is available for researchers to identify the optimal combination of therapeutics for treating GBM. Further, *Shigella* contains the equipment to ensure that therapeutic proteins are delivered directly into the cytosol of infected GBM cells via a type three secretion system (12, 20). In terms of safety, the potential for large cassette sizes allows for improved safety controls, as bacterial therapeutics factories can be programmed to self-destruct and/or suicide switches can be added ab lib into the platform (22, 24, 27). Finally, GBM-infecting *Shigella* remains sensitive to beta-lactams, so simple administration of penicillin or other blood–brain barrier-permeable antibiotics, including additional beta-lactams, vancomycin, aminoglycosides, fluoroquinolones (particularly moxifloxacin and levofloxacin), doxycycline, or polymyxins, can serve as master suicide switch(es) to immediately halt GBM-infecting *Shigella* activity in the case of adverse events such as fever or general infection (42). It is important to note that despite selecting a strain of *Shigella* with high sensitivity to antibiotics, it is possible that GBM-infecting *Shigella* could potentially become antibiotic-resistant, impacting the safety of our proposed drug delivery platform.

This study focused on developing *Shigella* to internalize into GBM cells as a foundation for a drug delivery system. We envision this platform serving as a tool for neurosurgeons to clean the margins of a GBM post-resection. GBM-infecting *Shigella* could be administered into the surgical cavity before closing to infect and eradicate cancerous cells in the invasive margin that cause disease recurrence. This approach bypasses the need for systemic administration and reduces the potential for internalization into off-target cells in the body. However, many steps are required before GBM-infecting *Shigella* are suitable for a drug delivery platform including stabilizing genetic material, controlling the bacterial population, ensuring intracellular replication, modulating immunogenicity, and weaponizing the system with cytotoxic proteins such as ribosome toxins (gelonin), cytokines (IL-2 and TNF), and autophagy-inducing proteins (caspases) (43–46). Additionally, further understanding of GBM-infecting *Shigella* on the tumor microenvironment including replication, macrophage polarization, and innate inflammation is essential to translating this novel platform into a drug delivery system.

Our data indicate that a myristoylated factor mediates the selective internalization of GBM-infecting *Shigella* into GBM cells. Removal of MsbB1 and MsbB2 myristoylation enzymes completely abrogates the internalization of GBM-infecting *Shigella* in GBM cells. Previous reports indicate that removing MsbB genes does not impair the internalization of *Shigella* in intestinal cells, the typical target of wild-type *Shigella* or *Salmonella* Typhimurium invasion into tumor cells (47–49). Further, a group reported removal of a single MsbB does not impair *Shigella* internalization in HeLa cells, and double knock-out only reduces internalization by 50% (50). Our data indicate ~75% reduction in internalization with single MsbB and 100% reduction (complete) of internalization with double MsbB knock-out, indicating that this mechanism may be unique for GBM-infecting *Shigella* to internalize in GBM cells (50). Our data are congruent with those of previous studies that removed MsbB genes to “detoxify” LPS. These studies indicated that MsbB enzymes interact with late acyltransferases to affect the branching of LPS to reduce the innate immunogenicity of LPS by lowering TLR4 engagement (35, 51–54). We also observed reduced TLR4 activity in MsbB knock-outs, and the sequencing data indicate the removal of only those two enzymes. Further, GBM cells that did internalize MsbB1 KO *Shigella* did so at a similar rate to R50 GBM-infecting *Shigella*, indicating that this effect is not a function of the reduced growth rate observed with MsbB knock-outs. Thus, we conclude that myristoylation (presumably to anchor a factor that mediates internalization to the bacterial membrane) is essential for GBM-infecting *Shigella* to internalize into GBM cells. However, our data do not indicate what that factor is. Whole genome sequencing did not identify obvious mutations in MsbB genes or a known myristoylated protein; however, future RNA-sequencing, epigenetic, and/or proteomic studies of *Shigella* membrane-bound proteins could identify factors with differential copy number, alterations in promoters, and/or *de-novo* localization to bacterial membranes that are driving the observed changes in *Shigella* internalization in GBM cells. These studies can be narrowed bioinformatically by eliminating proteins of interest that do not contain an N-terminal glycine (either directly after synthesis or after post-translational cleavage) that is required for modification with myristic acid (55). Identifying this factor could allow for the deletion of enzymes upstream of MsbB that still modulate LPS TLR4 engagement without abrogating internalization in GBM cells. Controlling innate immunogenicity could complement typical anti-cancer strategies such as arming GBM-infecting *Shigella* with immunomodulators, toxins, or other cytotoxic factors to increase the selective cytotoxic potential of this platform. Finally, other groups have utilized bacteria as a therapeutic agent to treat cancer, implying that this could be translatable to the clinic in the future when coupled with the correct cytotoxic payloads and safety measures (43, 56, 57). Finally, identifying these factors will be essential to understanding the mechanism of how GBM-infecting *Shigella* is preferentially internalizing in GBM cells compared to normal glia and neurons. Studies of GBM cells indicate abnormal patterns of glycosylation, lipids, and membrane proteins on the apical plasma membranes compared to non-malignant brain cells, which may interact with the myristoylated factor to provide the high specificity of internalization that we observed in this study (58–60).

In the future, we propose identifying GBM-infecting *Shigella* clones that preferentially internalize into a given patient’s GBM. As demonstrated in **Figure 2D**, we created an in-cell western to rapidly identify personalized GBM-infecting *Shigella* clones for a given patient tumor without further rounds of co-culture. This allows for the identification of clones that internalize into patient cell lines that cannot undergo multiple rounds of passaging or samples directly resected from the operating room. We propose expanding these studies to develop a pipeline capable of generating a personalized GBM-infecting *Shigella* therapy for each patient afflicted with a brain tumor. Additionally, weaponizing GBM-infecting *Shigella* with multiple modalities and safety controls in the future will allow testing the benefits of this platform compared to other microorganism strategies for treating brain tumors (43, 56). Finally, we propose identifying the myristoylated factor(s) driving the internalization of GBM-infecting *Shigella* into brain tumor cells to simultaneously improve the efficacy, specificity, and safety of our novel platform.

In conclusion, here, we presented the idea and proof-of-concept for generating GBM-infecting *Shigella* that selectivity internalizes into brain tumors compared to normal brain tissue. This platform demonstrates numerous potential benefits compared to existing microorganism platforms including high selectivity, unlimited cassette space, and the potential to improve the safety profile. Future studies with GBM-infecting *Shigella* will demonstrate the utility of this platform to benefit patients suffering from malignant brain tumors.

## Data availability statement

The datasets presented in this study can be found in online repositories. The names of the repository/repositories and accession number(s) can be found in the article/**Supplementary Material**.

## Ethics statement

Ethical approval was not required for the studies on humans in accordance with the local legislation and institutional requirements because only commercially available established cell lines were used. Ethical approval was not required for the studies on animals in accordance with the local legislation and institutional requirements because only commercially available established cell lines were used.

## Author contributions

AS designed assays and wrote the manuscript, GF designed assays and edited the manuscript, BD conceptualized the study and edited the manuscript, and BU conceptualized the study, designed assays, and wrote the manuscript. All authors contributed to the article and approved the submitted version.
