# Supplementary material for: Generating Shigella that internalize into glioblastoma cells
Source: Front Oncol. 2023 Nov 23;13:1229747. doi: 10.3389/fonc.2023.1229747 (PMC10710169; doi:10.3389/fonc.2023.1229747)
Supplement: Supplementary file 1 [file DataSheet_1.pdf]

Supplemental Figures:

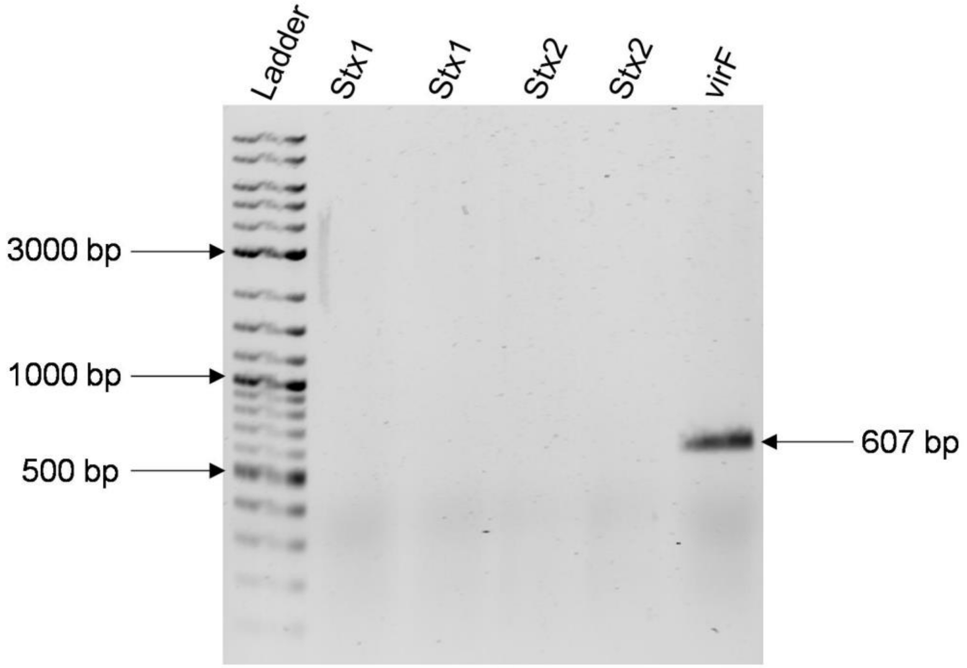

Supplemental Figure 1: Gel Images from Stx1 and Stx2 Detection: PCR is used to determine the presence of Shiga toxin 1 (Stx1) and Shiga toxin 2 (Stx2) genes in the starting strain of shigella used to create GBM-infecting shigella (Stx1=699 bp, Stx2=627 bp, virF= 607 bp).

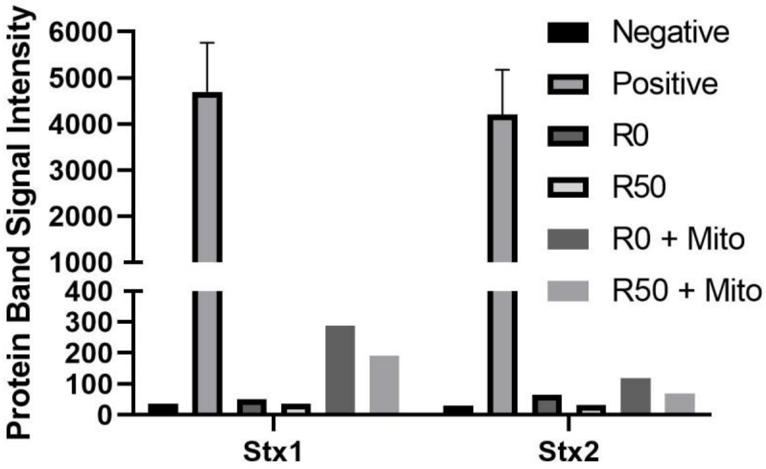

Supplemental Figure 2: The absence of Shiga toxin protein in GBM-infecting and non-GBM infecting Shigella (R50 and R0, respectively) is measured using a Shiga toxin detection kit. Shiga toxin was tested under normal growth conditions and under Mitomycin C (Mito) induction. Band intensity corresponding to Stx1, Stx2, or controls are plotted for each group.

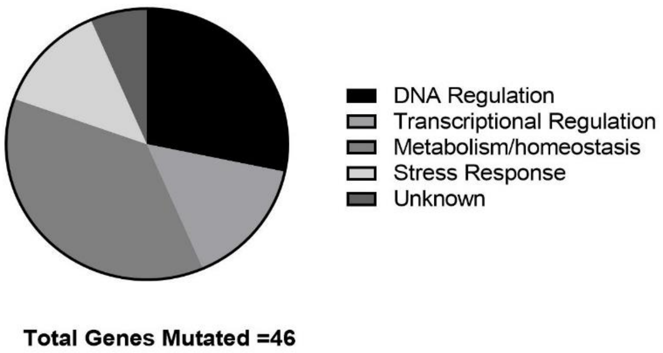

Supplemental Figure 3: We sequenced the genome of GBM-infecting Shigella to identify mutations that arose during engineering. These mutations were located inside 46 genes. Seventeen of the effected genes are related to metabolism/homeostasis, 13 related to DNA regulation, 7 related to transcriptional regulation, and 6 related to stress response.

Supplemental Table 1:

Gene	Name
S_RS27100	IS3 family transposase
WP_000125617.1	PTS maltose transporter subunit IICB
WP_000129530.1	bifunctional ADP-dependent NAD(P)H-hydrate dehydratase/NAD(P)H-hydrate epimerase
WP_000130034.1	D-alanine--D-alanine ligase
WP_000156579.1	helix-turn-helix transcriptional regulator
WP_000354046.1	FAD assembly factor SdhE
WP_000454701.1	TerC family protein
WP_000570673.1	ribosome-dependent GTPase TypA
WP_000577615.1	o-succinylbenzoate--CoA ligase
WP_000695507.1	alpha-glucosidase
WP_000741619.1	phosphate ABC transporter permease PstC
WP_000844882.1	primosomal replication protein N''
WP_000847163.1	aminotransferase class I/II-fold pyridoxal phosphate-dependent enzyme
WP_000850244.1	dimethylsulfoxide reductase subunit A
WP_000883129.1	NO-inducible flavohemoprotein
WP_000937883.1	exodeoxyribonuclease VII large subunit
WP_000983717.1	iron/manganese ABC transporter ATP-binding protein SitB
WP_001162179.1	metalloprotease PmbA1
WP_001194727.1	cell division protein ZapD
WP_001207893.1	nitrate/nitrite transporter NarU
WP_001223145.1	tRNA/rRNA methyltransferase
WP_001237077.1	glucosamine-6-phosphate deaminase
WP_001250207.1	peptide ABC transporter substrate-binding protein SapA
WP_001258671.1	aspartate--tRNA ligase
WP_001278539.1	FAD-dependent oxidoreductase
WP_001289665.1	electron transport complex subunit RsxE
WP_005049126.1	IS110-like element ISSfl4 family transposase
WP_011110552.1	ChiQ/YbfN family lipoprotein
WP_011110568.1	bifunctional acid phosphatase/4-phytase
WP_011110582.1	Gfo/Idh/MocA family oxidoreductase
WP_011110619.1	biosynthetic arginine decarboxylase
WP_011110635.1	IS66-like element ISSfl3 family transposase
WP_024260057.1	ATP-binding cassette domain-containing protein
WP_094081518.1	IS3-like element IS600 family transposase
WP_094092384.1	IS3-like element IS600 family transposase
WP_094105670.1	IS3-like element IS911 family transposase
WP_094105677.1	transposase
WP_094105679.1	IS3-like element IS911 family transposase
WP_167389526.1	ribosomal slippage
WP_171525780.1	IS3 family transposase
WP_240532658.1	ribosomal slippage
WP_240532662.1	ribosomal slippage
WP_276282982.1	P-type conjugative transfer protein TrbL

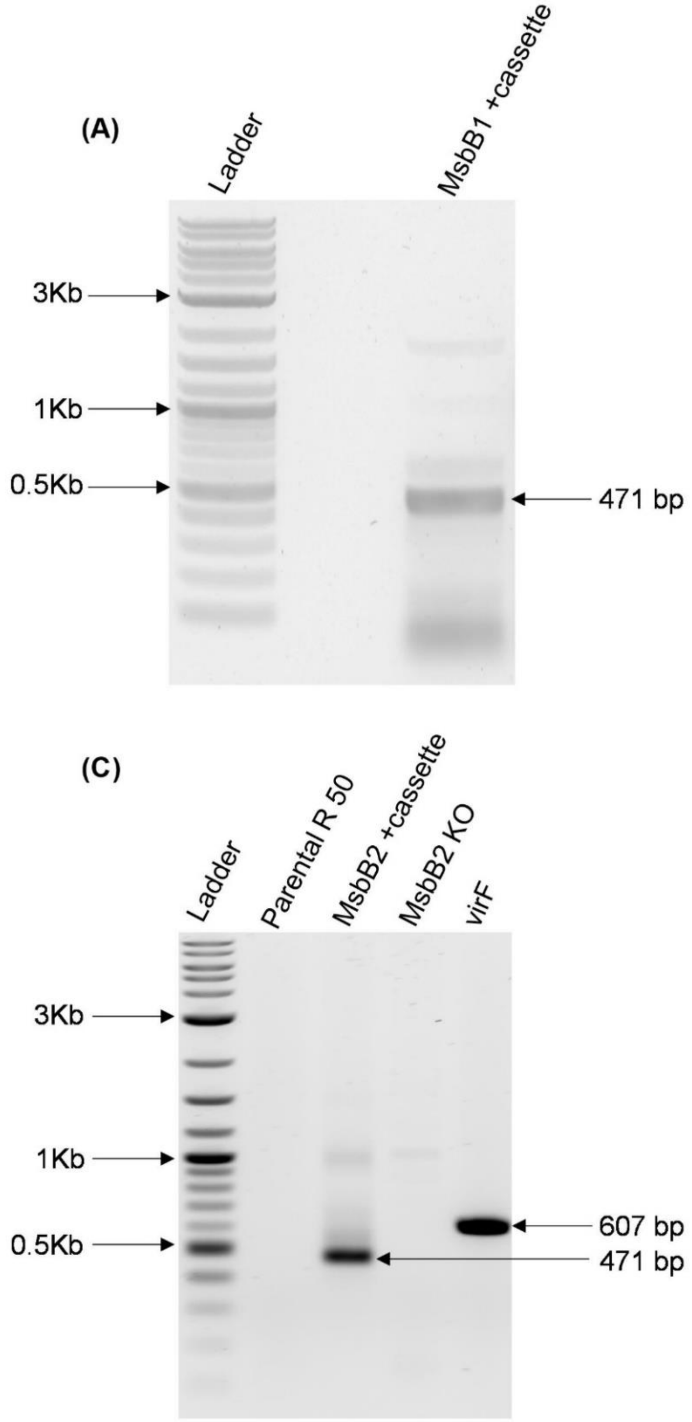

Supplemental Figure 4: Gel Images from MsbB Deletion: PCR is used to determine the presence of MsbB1 KO cassette (471 bp). **A)** Gel image of MsbB1 KO. **B)** MsbB2 KO gel comparing parental round 50 GBM-infecting Shigella to MsbB2+ cassette (471 bp), and **C)** MsbB2 KO with virF (607 bp) to ensure retention of virulence plasmid.

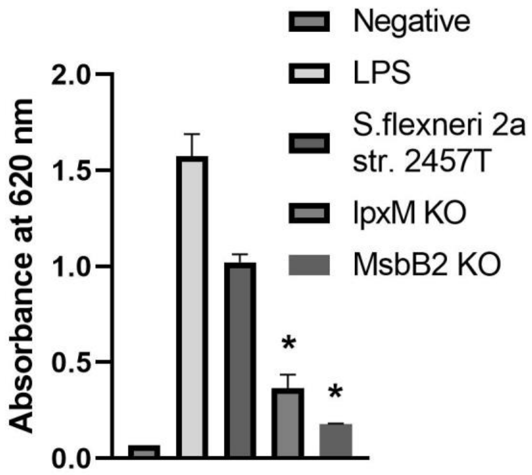

Supplemental Figure 5: TLR4 stimulation levels from *S. flexneri* and Shigella lpxM deletion. The optical density from this live cell assay is measured at 620 nm to quantify stimulation of TLR4.

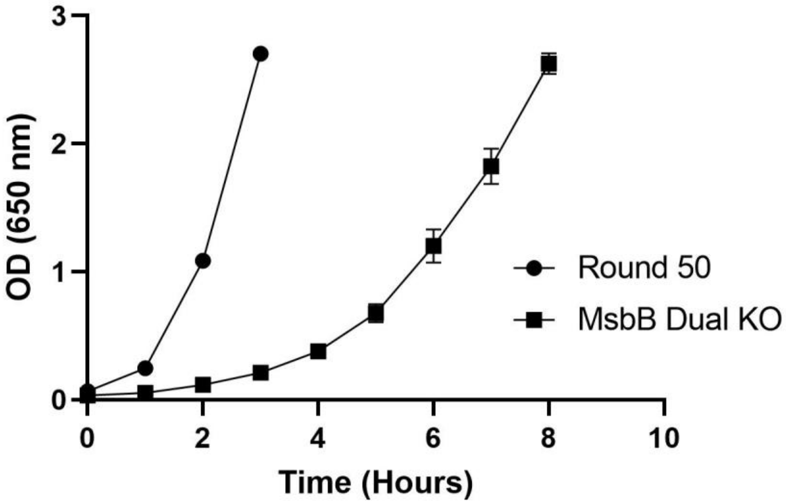

Supplemental Figure 6: Growth rate of round 50 shigella compared to MsbB Dual KO. The OD_{650} of each sample was measured every hour.
